# Weight changes and mobility in the early phase after hip fracture in community-dwelling older persons

**DOI:** 10.1007/s41999-020-00342-x

**Published:** 2020-06-16

**Authors:** Hanne Rosendahl-Riise, Jutta Dierkes, Svanhild Ådnanes, Vilde Aabel Skodvin, Elin Strand, Anette Hylen Ranhoff

**Affiliations:** 1 Mohn Nutrition Research Laboratory, Department of Clinical Medicine, University of Bergen, Bergen, Norway; 2 Center for Nutrition, Mohn Nutrition Research Laboratory, Department of Clinical Medicine, University of Bergen, Bergen, Norway; 3 Laboratory Medicine and Pathology, Haukeland University Hospital, Bergen, Norway; 4 Center for Nutrition, Department of Clinical Medicine, University of Bergen, Bergen, Norway; 5 Department of Clinical Science, University of Bergen, Bergen, Norway

**Keywords:** Nutritional status, Hip fracture, Mobility and sarcopenia

## Abstract

**Purpose:**

Hip fractures in older persons are associated with reduced mobility and loss of independence. Few studies address the nutritional status and mobility in the early phase after hip fracture. The objective of the present study was, therefore, to investigate weight changes and their effect on mobility during the first two months following hip fracture in community-dwelling older persons without dementia.

**Methods:**

Patients (> 60 years) admitted for a first hip fracture were recruited from two tertiary referral hospitals in Bergen, Norway. The patients' weights and dietary intakes were determined in the hospital and at home after two months. Mobility was assessed based on the New Mobility Score (NMS) (scale 0–9, with values > 5 regarded as sufficient mobility).

**Results:**

We included 64 patients (median age 80 years, 48 women, 16 men) with information on weight collected in the hospital. Follow-up measurements were available for 32 patients, corresponding to an attrition rate of 50%. The patients had a median weight loss of 1.8 kg (IQR = − 3.7, 0 kg). Most of them had reduced mobility at two months after the surgery [median NMS = 5 (IQR = 3–6)]. Both age and the weight change after surgery were predictors of the NMS at follow-up.

**Conclusion:**

Bodyweight loss was observed in three out of four patients in the early phase after hip fracture and was associated with decreased mobility measured by the NMS. The results should be interpreted with caution as half of the patients dropped out of the study and did not participate in the follow-up visit.

## Key summary points

**Aim:**

To investigate body weight changes and their effect on mobility during the first two months following a hip fracture.

**Findings:**

The loss of body weight was observed in three out of four patients in the early phase after hip fracture and was associated with decreased mobility measured by the NMS.

**Message:**

Bodyweight loss is common and may further reduce mobility in hip fracture patients, but these findings need more research.

## Background

Hip fractures are a major cause of disability in older persons and are associated with a high degree of long-term dependence and mortality [1, 2]. The need for care services after the fracture is associated with the patients’ age [3], functional status pre-fracture [4], cognitive impairment [5], and residency (community-dwelling versus institutions) [6]. The worst outcomes are observed among persons with both severe disabilities and cognitive impairment [1]. However, even persons without a diagnosis of cognitive impairment and community-dwelling before the fracture may not regain complete pre-fracture mobility within 1 year [4].

Many studies have shown that the average body mass index (BMI) is low in hip fracture patients, and malnutrition among them is common [7–9]. Owing to their increased energy requirements due to increased metabolism within the first months after the trauma [10], and to commonly low dietary intake after the fracture, these patients are at risk of further weight loss. However, this has not been studied thoroughly and concerning functional outcomes.

A wide range of tools is available for measuring functional outcomes, which makes it difficult to compare studies. The New Mobility Score (NMS) [11] is a simple questionnaire measuring environmental mobility and has frequently been used in hip fracture patients [12, 13]. The NMS has been compared with other tests and is comparable to Barthel-20 and Barthel-100 in hip fracture patients to predict survival, maintenance of residence status, and walking mobility [14]. The questionnaire has been validated and is not dependent on the observer [15]. It is thus a suitable and reliable instrument for assessing mobility in hip fracture patients.

Any weight loss is associated with loss of both fat mass and fat-free mass. It has been estimated that during intended weight loss, about 1/5 of the weight loss is fat-free mass [16]. In older persons, loss of muscle mass will increase the risk of sarcopenia or worsen established sarcopenia. Loss of weight and muscle mass may be associated with functional outcomes in hip fracture patients, but few studies have investigated this association.

The present study aimed to investigate the role of dietary intake and body weight change on mobility status in the early phase after hip fracture in community-dwelling patients without disability or dementia before the fracture.

## Methods

### Patient recruitment

Patients were recruited to this study from the orthopedic wards at Haukeland University Hospital and Haraldsplass Deacon Hospital, both located in Bergen, Norway (*n* = 50 and *n* = 14, respectively). A researcher visited the relevant departments on workdays to check for eligible participants.

Men and women aged over 60 years were eligible if they had been admitted for a first hip fracture, did not use walking aids and were community-dwelling before the fracture, and had normal cognitive function (as evaluated by the nurses in charge of the patients). Also, information on body weight was a requirement for eligibility.

Discharge from the hospital was in most of the patients to a rehabilitation unit in a specialist nursing home for about 2–3 weeks before they have been sent home. The patients were visited in their homes approximately two months after the surgery. Trained clinical dieticians performed all anthropometric measurements and dietary assessments. The two visits are hereby referred to as baseline and follow-up.

### Study procedures

At each of the visits, information was gathered on the body weight and dietary intake during the last 24 h by a structured 24 h recall. In the hospital, the patients' medical history and lifestyle habits were documented, and blood samples were collected. The visit at home included also measurements of height, handgrip strength (HGS), and body composition by bioelectrical impedance, and the NMS score was evaluated.

The 24 h recalls were following the USDA interview guidelines (automated multiple-pass method) [17]. Portion sizes were estimated with the aid of a booklet showing four different portions, or estimated in household measurements or from the number of items consumed. The data were entered in the online dietary tool ‘Kostholdsplanleggeren.no’, which is based on the official Norwegian food composition table and edited by the Norwegian Food Safety Authority and Norwegian Directorate of Health (https://www.kostholdsplanleggeren.no/).

### Baseline

This visit was usually done 2 days after surgery [median, (range 1–10)]. Bodyweight was measured while the patients wearing light hospital clothes and no shoes using a chair scale, (model 952, SECA, Hamburg, Germany).

Information on blood levels of hemoglobin, and serum levels of albumin, hs-CRP, and creatinine were collected from the electronic patients’ records (DIPS, Distributed Information and Patient System for hospitals), in most cases obtained the day before surgery. Comorbidities, medication, duration of stay in the hospital, type of the fracture, and type of the surgery were also established from patients’ records.

### Follow-up

Bodyweight was measured using an electronic flat scale, (SECA; model 877, Hamburg, Germany), while the patients were wearing ordinary clothes but no shoes. Heights were measured with a stadiometer (SECA, model 217, Hamburg, Germany), to the nearest 0.5 cm. From the weight and height measurements, the BMIs (kg/m^2^) was calculated.

The body composition was determined using single-frequency bioelectric impedance analysis (BIA) (50 kHz tetrapolar BIA 101 Anniversary Sport Edition, AKERN). The measurement was usually performed on the non-dominant side of the body unless the patients had a fistula on that side of the body. All jewelry, wristwatches, and belts were removed. The patients were usually non-fasting. The current "injection" electrode was placed on the dorsum of the hand, just above the phalangeal-metacarpal joint, and on the ventral side of the foot just below the transverse arch. Detector electrodes were placed on the dorsal side of the wrist, midline and in line with the pisiform bone, and across the ankle in line with the medial malleolus. The BIA measurements were not performed in patients with a pacemaker or an implantable cardioverter-defibrillator. Resistance and reactance values were obtained in Ohms, in addition to the phase angle. The formula of Kyle et al. [18] was used to calculate the skeletal muscle index (SMI), based on the appendicular lean mass (ALM/ht^2^). The BIA method has been validated for hip fracture patients [19].

The resting metabolic rate (RMR) was estimated using the Mifflin-St Jeor formula [20] and the ratio of reported energy intake to the RMR was calculated.

HGS was measured in triplicate, using a hydraulic hand dynamometer (JAMAR, Sammons Preston, Bolingbrook, IL, USA). The average of the three measurements was recorded. The NMS was used to assess mobility. The NMS is a composite score, reflecting the patient’s ability to walk indoors, walk outdoors, and go shopping alone, with or without an aid, which is defined as a cane, crutches, or a walker [11, 12]. The questions are answered using a score of 0, 1, 2, or 3 points. The range of the results is 0–9, and scores > 5 are regarded as showing sufficient mobility. 24 h recall dietary intake assessments were carried out as for the baseline visit.

### Statistics

Characteristics at baseline (age and sex distribution of the two hospitals, self-reported or measured body weight) were compared either using the Mann–Whitney *U* test and the chi-squared test.

The body weights, dietary intakes, and other study outcomes were compared between baseline and follow-up by the Wilcoxon rank-sum test for related samples.

The NMS determined at follow-up was used in a logistic regression analysis as a dichotomous outcome variable (NMS > 5 reflecting sufficient mobility and NMS ≤ 5 reflecting impaired mobility). Age was used as the explaining variable in all models, and only one additional explaining variable was used concurrently owing to the small number of patients.

Statistical software SPSS for Windows version 25 (IBM, NY, USA) was used for all calculations, *p* < 0.05 being taken as the threshold of statistical significance.

## Results

### Patients’ characteristics

An overview of the study participants is provided in Table 1. In the hospital, 64 patients (48 women, 16 men) agreed to participate in the study, and either reported their body weight (*n* = 30) or were weighed (*n* = 34). Half of the patients were older than 80 years. All had a low-energy fracture (54% a femoral neck fracture and 46% a pertrochanteric fracture); the method of surgery was osteosynthesis in 68%, hemiprosthesis in 29%, and total prosthesis in 3%. The median duration of stay in the hospital was 6 days (range 3–15). Laboratory tests showed albumin, hemoglobin, CRP, and creatinine levels within the reference range for most of the patients (Table 1). More than half of the women and 43% of the men had never smoked. The patients from the two hospitals did neither differ in age or weight at baseline, nor the sex distribution. There was also no difference whether the body weights were self-reported or measured (data not shown). Most patients were discharged to a rehabilitation unit (which in Norway is associated with a specialist nursing home), where they usually remained for two to three weeks.

**Table 1:** Baseline characteristics of the hip fracture patients

	All patients (*n* = 64)	Women (*n* = 48)	Men (*n* = 14)
Age (years)	81 (72, 86)	81 (72, 87)	80 (74, 82)
Length of stay (days)	6 Range: 3–15	6 Range: 3–15	6 Range: 4–14
Smoking status (*n* = 55) (*n*)			
Current	12	7	5
Ex-smoker	7	4	3
Never smoked	36	30	6
Missing information	9	7	2
Living alone (*n* = 61)	25 (41%)	21 (47%)	4 (25%)
Albumin (g/L)	41 (39, 44)	41 (39, 43)	42 (40, 44)
CRP (mg/L)	3 (1, 9)	4 (1, 16)	3 (1, 8)
Haemoglobin (g/L)	12.8 (11.3, 13.7)	12.6 (11.2, 13.6)	13.1 (12.4, 14.9)
Creatinine (µmol/L)	66 (56, 85)	63 (53, 78)	79 (64, 96)
Bodyweight (kg)	60.5 (54.6, 70.0)	58.5 (50.2, 67.8)	70.0 (60.8, 88.1)
Bodyweight assessment: Self-reported	30 (49%)	21 (43%)	9 (60%)
BMI (kg/m^2^)	22.7 (20.5, 25.4)	22.7 (19.9, 25.1)	22.8 (20.9, 26.7)
24 h recall at Days since surgery	2 Range: 1–10	2 Range: 1–10	2 Range: 1–7
Energy intake (kcal/day)	1314 (936, 1620)	1195 (918, 1468)	1562 (1235, 1927)
Energy intake (kcal/kg BW)	19.4 (14.1, 24.8)	19.0 (13.5, 24.9)	21.5 (15.7, 24.8)
Protein intake (g/day)	54 (37, 64)	47 (28, 61)	61 (59, 84)
Protein intake (g/kg BW)	0.84 (0.49, 1.07	0.80 (0.45, 1.02)	0.86 (0.71, 1.04)

All data are medians with interquartile range. Blood samples were collected prior to surgery. Comparisons between men and women were tested by the Mann–Whitney *U* test for continuous variables and by the chi-squared test for categorical variables. Energy intake was measured after surgery

*Chi-squared test

### Study-specific measures

The median weight was 58.5 kg (IQR = 50.2, 67.8) in the women and 70.0 kg (IQR = 54.6, 70.0) in the men (*p* < 0.05). The calculated BMI indicated underweight (according to WHO classification [21]) in 9.5% of the cases, weight within the normal range in 63%, and overweight in 25%; 3.1% of the patients were obese. The median reported energy intake at baseline was 1314 kcal/day (IQR = 936, 1620), with significant differences between the men and the women. However, when the energy and protein intakes were calculated per kg body weight (BW), the difference between the two sexes was no longer statistically significant (Table 1). In both men and women, the protein and energy intakes were lower than recommended [22, 23].

### Follow-up

In total, 32 patients agreed to receive a follow-up visit. This visit was at median 66 days after surgery (IQR = 60, 82) (Table 2). Bodyweight measurements revealed weight loss in 21 patients. The average weight loss was independent of whether the body weight at baseline had been measured or self-reported (*p* = 0.616). The reported energy intake was at the median 1.30 times the estimated resting metabolic rate (IQR = 0.88, 1.54), and the energy intake per kg BW was lower than recommended. The protein intake was low, especially in the women, and below the value recommended for older persons [22].

**Table 2:** Characteristics of patients at the follow-up visit (all patients and women: medians with IQR, men: range)

	All patients *n* = 32	Women *n* = 24	Men *n* = 8
Age (years)	76 (69, 87)	77 (69, 88)	75 (61–95)
Days since surgery	66 (60, 82)	69 (60, 84)	65 (38–83)
Weight (kg)	60.6 (49.6, 66.9)	55.5 (49.2, 64.7)	68.6 (48.3–91.8)
Weight change (kg) since surgery	− 1.8 (− 3.7, 0)	− 1.4 (− 2.6, 1.9)	− 3.2 (− 6.0 to 2.9)
Energy intake (kcal/d)	1511 (1119, 1766)	1473 (1096, 1635)	1676 (940–2369)
Energy intake (kcal/kg BW)	25.0 (17.5, 32.9)	24.5 (18.2, 33.4)	29.4 (10.2–36.0)
Protein intake (g/d)	54 (41, 73)	52 (40, 71)	68 (38–116)
Protein intake (g/kg BW)	0.88 (0.73, 1.30)	0.88 (0.74, 1.30)	1.09 (0.53–1.76)
New mobility score	5 (3, 6) Range: 0–9	4 (3, 6) Range: 0–9	5 Range: 2–6
SMI (ALM/ht^2^)	5.6 (5.1, 6.5)	5.4 (4.9, 5.9)	7.4 (5.9–7.8)
Handgrip strength (kg)			
Mean, right hand	18 (13, 26)	17 (12, 21)	32 (18–40)
Mean, left hand	17 (12, 26)	14 (11, 19)	31 (25–40)

Appendicular lean mass (ALM) was at the median 14.4 kg in the women and 20.7 kg in the men. The median SMI (ALM/ht^2^) was 5.4 kg/m^2^ in the women and 7.4 kg/m^2^ in the men. The HGS measurements revealed low grip strength in the women (median on either side < 20 kg). The median HGS in the men was higher, exceeding 30 kg on both sides.

The median NMS point score was 5. The NMS and the HGS correlated significantly with each other (*r* = 0.35, *p* = 0.05), patients with low NMS also having significantly lower HGS.

### Logistic regression analysis with NMS as the outcome

At follow-up, 17 patients (13 women and 4 men) had NMS ≤ 5, and 14 (12 women and 2 men) had NMS > 5. The patients with scores ≤ 5 were older, lost more weight, had lower energy intakes in the hospital, and lower HGS. There was no difference in the sex distribution, days after surgery, duration of stay in the hospital, or the energy intakes at the follow-up (Table 3).

**Table 3:** Comparison of patients according to the NMS result (inadequate mobility (NMS ≤ 5) and sufficient mobility (NMS > 5) (medians with IQR)

	NMS ≤ 5 *n* = 17	NMS > 5 *n* = 14	*p*
Age (years)	79 (74, 89)	70 (63, 76)	0.003
Sex (M/W)	4/13	4/10	0.75
Living alone/with partner *n* = 29	6/9	4/10	0.52
Days since surgery			
Baseline	3 (2, 5)	2 (1, 3)	0.200
Follow-up	65 (60, 70)	70 (54, 84)	0.591
Length of stay (days)	6 (5, 8)	5 (5, 9)	0.356
Weight at baseline (kg)	57.2 (50.0, 67.0)	67.4 (54.4, 69.9)	0.399
Weight at follow-up (kg)	54.2 (49.0, 63.6)	66.6 (57.8, 68.2)	0.053
Weight change (kg)			
Since surgery	−2.6 (−5.3, −0.7)	−1.2 (−2.3, 3.0)	0.032
Energy intake (kcal/day)			
Baseline	1331 (632, 1616)	1547 (1317, 1956	0.084
Follow-up	1421 (985, 1703)	1615 (1131, 1896)	0.246
Energy intake (kcal/kg/BW)			
Baseline	17 (9, 24)	26 (22, 29)	0.029
Follow-up	26 (16, 33)	26 (17, 33)	0.822
Protein intake (g/day)			
Baseline	56 (25, 65)	62 (49, 85)	0.065
Follow- up	48 (37, 67)	63 (47, 80)	0.092
Protein (g/kg BW)			
Baseline	0.7 (0.4, 1.1)	1.1 (0.9, 1.2)	0.059
Follow-up	0.9 (0.7, 1.1)	1.0 (0.7, 1.5)	0.473
Handgrip strength (kg), right-hand side*	17 (11, 20)	23 (16, 32)	0.046
SMI (ALM/ht^2^)* *	5.4 (5.1, 5.9)	6.1 (5.2, 7.3)	0.134

Continuous variables were tested with the Mann Whitney *U* test, and categorical variables were tested with Chi-square test

*Left-hand side not shown, but similar results

**BIA measurements were available in 24 patients

Logistic regression with NMS ≤ 5 and > 5 as outcomes revealed that only age was significantly associated with the NMS. A weight loss of 1 kg increased the risk of NMS < 5 by 39% (95% CI − 3%, 98%). The other variables tested (HGS, energy intake at home, SMI) were not associated with NMS as a dichotomous variable. This result remained substantially unchanged when comorbidities (cardiovascular disease, type 2 diabetes mellitus, chronic obstructive pulmonary disease (COPD), cancer, hypertension, stroke), method of surgery, fracture site, sex, days to the follow-up visit, energy intake at the baseline, protein intake at the baseline, energy intake at follow-up visit or protein intake at follow up (total protein, g/kg BW) were entered one by one into the model.

## Discussion

The principal finding of this observational study is that in hip fracture patients two months after the fracture, reduced mobility (NMS ≤ 5) was associated with age and moderate weight loss. These associations were independent of other factors, including sex, dietary energy or protein intake, and major comorbidities (cardiovascular disease, type 2 diabetes mellitus, hypertension, COPD). The observed body weight change was less than 5% of initial body weight in most cases, and, therefore, would not be considered as a clinically significant weight loss (which is usually set at 5%) [24]. Thus, even small weight losses may have a clinical meaning in these patients which is line with previous investigations both by our group and others [25–27].

### Physical function and mobility

The NMS was used for the assessment of mobility. This score can easily be established, as it is based on three questions, is self-reported, and is reliable in hip fracture patients [12, 13, 28]. Furthermore, the NMS measures environmental mobility and does not rely on physical performance, which makes it suitable for hip fracture patients even shortly after the surgery. The NMS score can be regarded as a valid outcome, and its overall good agreement with other established, but more complex instruments have been reported [14]. Studies by other investigators have shown that only about half of the patients have satisfactory mobility outcomes after two to three months [12], which is in line with our findings. However, early mobility after fracture is important for maintaining independence [29].

### Weight change, body composition, and functional measures

Few studies have investigated the bodyweight development and body composition in the early phase after a hip fracture. In longitudinal studies, loss of fat-free mass has been reported during the first few months and in the first year after the hip fracture [30–33]. Even fewer studies have linked changes in body composition and loss of fat-free mass to functional outcomes. In two longitudinal studies from Baltimore hip studies [30, 32], no association of changes in body composition with functional outcomes was observed. In a recent study from Norway [13], an association was found between sarcopenia (as defined by low HGS and ALM/ht^2^) and inadequate NMS after one year. Unfortunately, this study did not include weight changes.

Although many studies in hip fracture patients report body weights and/or the BMI, it often remains unclear whether the reported body weights were measured or self-reported [1, 34] illustrating the challenges of weight measurement in patients with a hip fracture which were also experienced in the present study. Also, few studies have reported measured weight during follow up. In the present study, there was no difference in weight loss between those who had weights measured or self-reported in the hospitals. To the best of our knowledge, there is only one other study [31], in which measured weights were reported. These authors also observed that patients who experienced the greatest weight loss showed reduced functional outcomes at 2, 6, and 12 months after the hip fracture surgery.

At first glance, it may be surprising that such a small weight loss is linked to physical function. In the present study, weight and BMI before the fracture were rather low, and further weight loss may mostly be fat-free mass, although this remains speculative as we did not measure body composition in the hospital. Indeed, we also observed an association between mobility and muscle strength, indicating that those with stronger muscle strength regained better mobility. Results of the present study suggest that patients’ weight should be closely monitored after a hip fracture, even though measures to prevent weight loss have not been thoroughly investigated.

### Dietary intake and nutritional status

Similarly to other authors [8, 9, 35–37], we observed low energy intakes in the patients throughout the study. Energy malnutrition seems to be common in hip fracture patients [8, 10, 38]. Since hip fracture patients are often lean [1] and show an increased metabolism [10], a low dietary intake gives cause for concern. We also observed very low energy intakes in the hospital, which were weakly associated with low NMS after two months. It is unknown whether the energy deficit could be overcome by oral nutrition supplements, and what would be the outcome of such a nutritional intervention. However, the use of ONS has been recommended for all orthopedic patients in the hospital already in 2006 [39], although based on limited evidence.

### Strengths and limitations of the study

The major strengths of the present study were the longitudinal design and the direct assessment of the patients instead of using proxies. All measurements and interviews were performed face to face, not by telephone. Few studies have investigated the early phase after hip fracture and included physical measurements of the patients, even though early recovery seems to be important for later outcomes [29, 40]. The heterogeneity that is present in hip fracture patients precludes a comparison of outcomes across studies. A major factor is the cognitive status of the patients [1, 12]. In the present study, only patients with normal cognitive function and ones who had been community-dwelling before the fracture were included, so that our results should only be compared with results in similar patients. Our results are in line with those of other studies using the NMS [12, 13]. The NMS questionnaire is cheap, reliable, and easy to perform, but on the other hand, is not informative on specific limitations of mobility.

We focused on weight rather than on the BMI, because height measurements may not be reliable in older persons owing to the shrinkage of height with advancing age [41].

Nevertheless, the study suffers from several limitations. In the first place, in about half of the patients, the bodyweight could not be measured because of the fracture, and in these cases, self-reported weights were documented. However, we did not observe any difference in weight changes according to whether the body weights had been measured or self-reported. Secondly, the recordings of dietary intakes may have been affected by underreporting, a common feature in dietary assessments.

Only patients who had been community-dwelling before and after the fracture and who did not have a diagnosis of dementia were included, i.e. the healthiest in the hip fracture population. Despite including only the healthiest of such patients, we observed substantial losses to follow-up, as the patients were either too sick, developed delirium or impaired cognitive function, or refused further participation. Those who declined further participation did not differ in age, weight at baseline, sex distribution, or length of stay in the hospital (data not shown). Unfortunately, we were unable to quantify the reasons for non-participation further. Overall, the attrition rate of 50% is in line with other studies in older persons [31, 33]

As the number of patients was relatively small, the results should be confirmed in future studies.

## Conclusion

In this study of community-dwelling hip fracture patients with normal cognitive function, it was observed that older age and body weight loss were associated with lower mobility outcomes after two months. The results have to be interpreted with caution as we observed high drop out during the study, and the study was rather small.

## Data Availability

The datasets generated and analyzed during the current study are available from the corresponding author on reasonable request.

## Abbreviations

ALM: Appendicular lean mass
BIA: Bioelectrical impedance analysis
BMI: Body mass index
BW: Body weight
COPD: Obstructive pulmonary disease
HGS: Handgrip strength
NMS: New Mobility Score
REC: Regional Ethical Committee
RMR: Resting metabolic rate
SMI: Skeletal muscle index
